# Differential Impact of the Pinewood Nematode on *Pinus* Species Under Drought Conditions

**DOI:** 10.3389/fpls.2022.841707

**Published:** 2022-03-10

**Authors:** Mariana Estorninho, Sergio Chozas, Angela Mendes, Filipe Colwell, Isabel Abrantes, Luís Fonseca, Patrícia Fernandes, Catarina Costa, Cristina Máguas, Otília Correia, Cristina Antunes

**Affiliations:** ^1^Centre for Ecology, Evolution and Environmental Changes, Faculdade de Ciências, Universidade de Lisboa, Lisbon, Portugal; ^2^Infarm, Crop Science Team, Amsterdam, Netherlands; ^3^Centre for Functional Ecology, Department of Life Sciences, University of Coimbra, Coimbra, Portugal

**Keywords:** *Bursaphelenchus xylophilus*, climate change, physiological responses, pine wilt disease, *Pinus pinaster*, *Pinus pinea*, *Pinus radiata*, wilting symptoms

## Abstract

The pinewood nematode (PWN), *Bursaphelenchus xylophilus*, responsible for the pine wilt disease (PWD), is a major threat to pine forests worldwide. Since forest mortality due to PWN might be exacerbated by climate, the concerns regarding PWD in the Mediterranean region are further emphasized by the projected scenarios of more drought events and higher temperatures. In this context, it is essential to better understand the pine species vulnerability to PWN under these conditions. To achieve that, physiological responses and wilting symptoms were monitored in artificially inoculated *Pinus pinaster* (*P. pinaster*), *Pinus pinea* (*P. pinea*), and *Pinus radiata* (*P. radiata*) saplings under controlled temperature (25/30°C) and water availability (watered/water stressed). The results obtained showed that the impact of PWN is species-dependent, being infected *P. pinaster* and *P. radiata* more prone to physiological and morphological damage than *P. pinea*. For the more susceptible species (*P. pinaster* and *P. radiata*), the presence of the nematode was the main driver of photosynthetic responses, regardless of their temperature or water regime conditions. Nevertheless, water potential was revealed to be highly affected by the synergy of PWN and the studied abiotic conditions, with higher temperatures (*P. pinaster*) or water limitation (*P. radiata*) increasing the impact of nematodes on trees’ water status. Furthermore, water limitation had an influence on nematodes density and its allocation on trees’ structures, with *P. pinaster* revealing the highest nematode abundance and inner dispersion. In inoculated *P. pinea* individuals, nematodes’ population decreased significantly, emphasizing this species resistance to PWN. Our findings revealed a synergistic impact of PWN infection and stressful environmental conditions, particularly on the water status of *P. pinaster* and *P. radiata*, triggering disease symptoms and mortality of these species. Our results suggest that predicted drought conditions might facilitate proliferation and exacerbate the impact of PWN on these two species, through xylem cavitation, leading to strong changes in pine forests of the Mediterranean regions.

## Introduction

Pine wilt disease (PWD) results from the infection of *Pinus* species by the pinewood nematode (PWN), *Bursaphelenchus xylophilus* (*B. xylophilus*). Currently, *B. xylophilus*, a phytophagous and mycophagous nematode, is declared a major threat to pine forests worldwide (Braasch, 2008; Carnegie et al., 2018; European and Mediterranean Plant Protection Organization [EPPO], 2021). Once inside the plant, its feeding and rapid multiplication in the cortex and in resin ducts may lead to a series of biochemical (Pimentel et al., 2017; Gaspar et al., 2020), physiological (Fukuda, 1997), and histological (Myers, 1986) changes. The damage of the resin ducts by the nematode strongly constrains water conduction in the xylem, embracing a series of physiological alterations such as leaf water potential (Fukuda, 1997; Suzuki, 2002; Menéndez-Gutiérrez et al., 2018; Yazaki et al., 2018), transpiration, and photosynthesis decrease (Fukuda, 1997; Suzuki, 2002; Yazaki et al., 2018). These physiological changes are usually responsible for visible symptoms such as discoloring and wilting of needles, loss of resin exudation from the wounded bark, and at an advanced stage death (Mamiya, 1983; Woo et al., 2010; Zas et al., 2015; Menéndez-Gutiérrez et al., 2018). Nevertheless, the period from infection to appearance of symptoms and tree death varies with a combination of biotic and abiotic factors such as trees’ susceptibility, the number of nematodes transmitted by the insect vector, *Monochamus* spp., and climatic conditions (Mamiya, 1983; Menéndez-Gutiérrez et al., 2017).

Forest mortality, attributed to PWN infection, and the fast spread of PWD cannot be dissociated from the effects of climate, topography, and human activities (i.e., infected timber exports) (Tóth, 2011; Calvão et al., 2019). However, the success of its establishment is mainly related to the development of the nematode–host species interaction (Futai, 2013), anatomy and health conditions of host trees (Nunes da Silva et al., 2015; Zas et al., 2015), PWN virulence (Aikawa and Kikuchi, 2007; Filipiak, 2015), and climatic conditions (Kiyohara and Bolla, 1990; Wang, 2014). In particular, PWD dynamics and its impact on trees was shown to be highly dependent on different climatic conditions such as light intensity (Kaneko, 1989; Kanzaki et al., 2012) and temperature, with significant disease expression restricted to areas above the isotherm of 20°C (Rutherford and Webster, 1987; Soliman et al., 2012) and a lethal effect above 21°C (Sikora and Malek, 1991). Under higher temperatures, a faster dispersion and multiplication of PWNs inside pine seedlings (day, 30°C; night, 25°C) than at low temperatures (day, 25°C; night, 20°C) might occur (Ichihara et al., 2000). Temperature is also known to have a crucial role in nematode–vector interaction, with an increase in nematode transmission, and vector’s life span under 20–26°C (Jikumaru and Togashi, 2000; Ohsawa and Akiba, 2014), longer vector’s flight seasons, and higher nematode loads in warmer areas (Rutherford and Webster, 1987; Pimentel et al., 2014). At lower temperatures, the development of the symptoms is slower, with no disease development in infected trees maintained at 18°C or below, whereas at 30–32°C PWD incidence and tree mortality are highest and symptoms development is faster (Rutherford and Webster, 1987; Pimentel and Ayres, 2018). Besides temperature, several studies settled that dry conditions (i.e., low water availability) result in an increase in nematode population, faster PWD progress, advanced disease symptoms, and higher tree mortality rate (Mamiya, 1983; Ikeda, 1996; Braasch, 2000; Suzuki, 2002; Wang, 2014; Yazaki et al., 2018). However, there are still unanswered questions related to how high temperatures, low water availability, and their combined effect influence PWD development in pine species occurring in the Mediterranean region.

In the Mediterranean basin, where there is a strong seasonal drought period, with warm dry summers, and where pine forests are one of the major wood resources, PWD is a current important issue. Concerns regarding insect and pests outbreaks are further emphasized by the projected scenarios of increasing drought episodes and rise of temperature for this region (Intergovernmental Panel on Climate Change [IPCC], 2014; Anderegg et al., 2015b). In Europe, PWD was detected for the first time in Portugal in 1999 (Mota et al., 1999) later in Spain (Abelleira et al., 2011) and in Madeira Island (Fonseca et al., 2012). Its expansion to all Europe is a worry (Vicente et al., 2012), but, so far, the disease remains epidemic in a restricted area. In Portugal, its limits had extended since the first report causing a regression of pine forest (Mota and Vieira, 2008b; Vicente et al., 2012). These impacts are expected to be aggravated in the next years (De la Fuente and Beck, 2018) with forecasted losses over 80% of the total stock of coniferous trees in Portugal, leading to a negative economic and ecological impact (Soliman et al., 2012). The maritime pine, *Pinus pinaster* (*P. pinaster*) Aiton, is a thermophilic, light demanding, and fast-growing species whose plantation has increased due to its versatility. Nevertheless, is considered very susceptible to pests and diseases, being one of the most significant species affected by PWN in Europe (Vicente et al., 2012; Nunes da Silva et al., 2015; Pimentel et al., 2017). In addition, under the current and projected climatic context, other coexisting *Pinus* species might become more vulnerable to the nematode infection and consequently to PWD. This is particularly important for species that, although currently considered less susceptible to PWN, have an important role in the economy, such as stone pine, *Pinus pinea* (*P. pinea*) L. and radiata pine, *Pinus radiata* (*P. radiata*) D. Don (Mota and Vieira, 2008a; Mota et al., 2009; dos Santos and de Vasconcelos, 2012; Menéndez-Gutiérrez et al., 2018). Contrary to *P. pinaster* and *P. radiata*, *P. pinea* is considered a slow-growth species able to thrive in dry conditions and with a strong resistance to pests and diseases (Abad Viñas et al., 2016). Hence, contrasting susceptibility of these three species to PWN is expected to be related in one hand to their defenses (anatomical, constitutive, and chemical), enabling the tree to reduce the nematode’s ability to migrate and proliferate within the tree, and on the other hand to their drought resistance strategies, enabling the tree to be generally less vulnerable to external biotic pressure (Nunes da Silva et al., 2015; Pimentel et al., 2017; Menéndez-Gutiérrez et al., 2018).

In this context, it is essential to better understand the vulnerability of pine species to PWN under different scenarios of environmental conditions. Therefore, the main objective of this study was to evaluate the physiological impacts of PWN on different pine species under high temperatures and drought (low water availability) and to study their combined effect on the severity of nematode’s infection. To achieve that we investigated physiological responses and wilting symptoms of *P. pinaster*, *P. pinea*, and *P. radiata* to the interaction between nematode infection and two key environmental factors: temperature and water availability. We hypothesized that (i) physiological susceptibility to PWN will depend upon *Pinus* species, being the species considered more drought and nematode resistant the one that will show lower impacts of PWN on photosynthetic activity and water status, and (ii) the combination of higher temperature and water stress will enhance PWD development, with a greater and faster onset of physiological and morphological symptoms and higher tree mortality in all the studied pine species.

## Materials and Methods

### Pinewood Nematode Inoculum

The *B. xylophilus* isolate (BxPt17AS), a virulent PWN isolate (Cardoso et al., 2016, 2022; Pimentel et al., 2017; Silva et al., 2021), used in this experiment was obtained from infected maritime pine from Alcácer do Sal (Portugal). The isolate was established and maintained in cultures of *Botrytis cinerea* Pars. grown on malt extract agar medium and incubated at 25°C (Fonseca et al., 2012; Cardoso et al., 2016; Silva et al., 2021).

### Experimental Design

The study was conducted in 5-year-old plants of three pine species with similar intraspecific heights (*P. pinaster:* 2.05 ± 0.2 m; *P. pinea:* 1.61 ± 0.1 m; *P. radiata:* 1.32 ± 0.1 m). One month before the PWN artificial inoculation, 40 plants of each species were divided into two groups. One group was acclimated at 25°C and the other at 30°C in two separate compartments of a greenhouse at the Faculty of Science of the University of Lisbon. Climatic conditions were continuously monitored with HOBO^®^ data loggers (Onset, MA, United States). On the 25°C treatment, the maximum and minimum temperatures reached were 15.2 and 31.1°C and the mean daily temperature was 22.9 ± 4°C. The 30°C greenhouse had a daily mean temperature of 25.6 ± 7°C and the maximum and minimum temperatures registered were 16 and 40.1°C, respectively. In both cases, plants were under natural photoperiod and solar radiation, and the air relative humidity (RH) was preserved at 50–60%. During the trial, the plants were kept in pots with the soil of the forest nursery from where they came (Aliança, Grupo Portucel Soporcel, Portugal).

Seventeen days before the PWN artificial inoculation, two water regime treatments were added to each pine species at both temperatures: (i) watered (W, *n* = 10/species); and (ii) water stressed (WS, *n* = 10/species). To maintain the assigned treatments, soil volumetric moisture was regularly monitored with a ThetaProbe MLX2 (LAB-EL Laboratory Electronics, Poland) before watering, allowing to control and adjust irrigation during the experiment. In the W regime, the trees were watered, with tap water, three times a week and the WS plants twice a week. The mean soil water content in W plants was 0.17 ± 0.09 m^3^m^–3^, while in WS plants was 0.07 ± 0.08 m^3^m^–3^.

**FIGURE 1:**
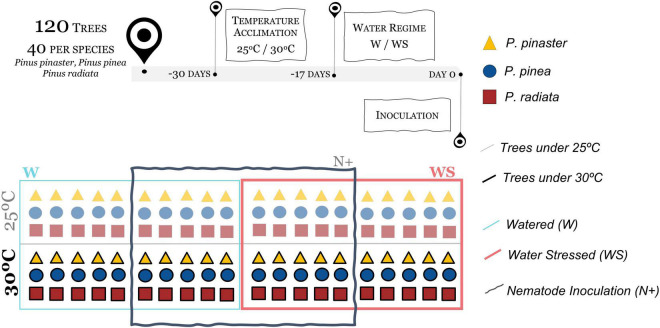
Conceptual diagram of the experimental setup. The upper panel represents the initial process, highlighting the treatments [temperature (25/30°C); water regimes (W – watered/WS – water stressed); *Bursaphelenchus xylophilus* inoculation (N– non-inoculated/N+ inoculated)] applied to the three *Pinus* species (*P. pinaster, P. pinea*, and *P. radiata*). The lower panel shows the eight treatments applied to a total of 120 trees, 40/*Pinus* species, five trees of each species/treatment (W25N–; W30N–; W25N+; W30N+; WS25N–; WS30N–; WS25N+; WS30N+).

To initiate the trial (day 0), eight treatments were established: (i) watered plants at 25°C non-inoculated (W25N−); (ii) watered plants at 25°C inoculated with PWN (W25N+); (iii) WS plants at 25°C non-inoculated (WS25N−); (iv) WS plants at 25°C inoculated with PWN (WS25N+); (v) Watered plants at 30°C non-inoculated (W30N−); (vi) Watered plants at 30°C inoculated with PWN (W30N+); (vii) WS plants at 30°C non-inoculated (WS30N−); and (viii) WS plants at 30°C inoculated with PWN (WS30N+) (Figure 1). The PWN inoculation of the trees (*n* = 5 × 3 × 4 = 60) in the treatments W25N+, WS25N+, W30N+, and WS30N+ was performed artificially. The trees (*n* = 5 × 3 × 4 = 60) of the control treatments (W25N−, WS25N−, W30N−, and WS30N−) were inoculated with sterilized water.

### Artificial Inoculation

For tree inoculation, a wound was made on the main stem of each plant by removing an area of bark (2 cm × 1 cm), and a piece of cotton was placed in the wound. Then, the nematode suspension, already adjusted with sterilized water to achieve a concentration of 6,000 (mixed developmental stages)/100 μL, or sterilized water was pipetted onto the cotton and covered with parafilm to prevent desiccation (Woo et al., 2008; Yazaki et al., 2018).

### Assessment of Plant Physiological Performance

Physiological measurements were taken twice a week in three (for gas-exchange measures) or five (for water potential and reflectance index) plants of the three *Pinus* species at each treatment. The sampling stopped when the individuals died or 75 days after inoculation.

#### Leaf Water Potential

Predawn leaf water potential (Ψ_pd_) was measured before sunrise with a Scholander pressure chamber (Manofrígido, Lisboa, Portugal). Water potential was assessed in 1-year-old needles in all plants.

#### Gas Exchange

Three plants of each pine species in each treatment were randomly selected, and gas-exchange measurements were performed between 10:00 a.m. and 12:30 a.m. with a portable infrared gas analyzer (GFS-3000, Walz, Effeltrich, Germany). Carbon assimilation (A, μmol m^–2^ s^–1^), leaf transpiration rates (E, mmol m^–2^ s^–1^), and stomatal conductance (gs, mmol m^–2^ s^–1^) were measured with an incident photosynthetic photon flux density (PPFD) of 1000 μmol m^–2^ s^–1^, a flow of 700 μmol/s, and under greenhouse CO_2_ concentration and RH conditions (in a leaf chamber of 8 cm^2^). The measures were taken in two needles of each tree and their specific leaf areas (accessed with a leaf area meter) were used to estimate respective A, E, and gs (and the mean value was considered for further analysis).

#### Photochemical Reflectance Index

Reflectance was assessed using a spectroradiometer (UNISPEC-SC Spectral Analysis System, PP Systems, United States) 15 days after the inoculation. The measurements were carried out in 5 needles/plant/treatment (mean value was considered for further analysis). Photochemical Reflectance Index (PRI) was calculated as PRI = (R531−R570)/(R531 + R570), being R531 and R570 reflectance at wavelengths of 531 and 570 nm, respectively. PRI is based on carotenoids estimation and is highly associated with photosynthetic light use efficiency (Peñuelas and Filella, 1998). Thus, it can be used as an index of photosynthetic activities in which low PRI values are associated with responses to stress factors such as high temperatures, water stress, excess of light exposition, salinity, and presence of pests (Wong and Gamon, 2015; Sukhova and Sukhov, 2018).

### Wilting Symptoms

To compare PWD development across species under different conditions over time, wilt symptoms were examined visually and recorded for all pine trees under the nematode treatments (W25N+; WS25N+; W30N+; WS30N+) and the control situation (W25N−). The symptoms considered were alteration of leaf color, from green to yellow and brown, followed by canopy foliage loss (Mamiya, 1983). These symptoms of needle dehydration were classified into a six class scale based on wilting and percentage of brown needles in the tree: 0 – tree without symptoms, I – <10% brown leaves, II – 10–50% brown leaves, III – 50–80% brown leaves, IV – >80% brown leaves, and V – dead tree without leaves (Proença et al., 2010). We considered the trial to be ended when tree mortality was verified within the water regime trial-set of 10 trees (i.e., in WS25N+//W25N+ or WS30N+//W30N+). After 75 days, even if there were plants still alive the assessment stopped.

### Pinewood Quantification

Pinewood nematode was quantified in plants inoculated with the nematode (N+, *n* = 60), by the end of each trial. We considered the trial to be ended when tree mortality was verified within the water regime trial set of 10 trees (in WS25N+//W25N+ or WS30N+//W30N+). Plants were removed from pots and the respective stem, branches, and roots separated. Each component of the plant was sliced into 5 mm thick, and nematodes extracted, using the tray method (Whitehead and Hemming, 1965), identified, and quantified under a stereomicroscope. After 75 days, all the plants still alive were also cut and the same procedure was applied.

### Statistical Analysis

Aiming at identifying physiological patterns among the species and treatments, and to reduce and integrate the physiological parameters, a principal component analysis (PCA) was performed based on all physiological variables (PRI, A, gs, E, and Ψ_pd_), using the mean (representative of the data set) and minimum (lowest physiological performance reached) of individual data measured throughout the experiment, using facto extra R package (Kassambara and Mundt, 2019). Individual coordinates of the axis that accounted for the highest percentage of variance explained (in this case the first axis of the PCA, PC1) were extracted and used in further statistical analysis.

The effects of the interaction Species*Inoculation* Temperature*Water Regime on PC1 (a synthesis trait for plants’ photosynthetic performance); the effects of the interaction of the treatments applied (Inoculation*Temperature*Water Regime) by species on (i) PC1 and (ii) Ψ_pd_ (a parameter not integrated in the PC1, reflecting plant water status); and the effects of the interaction Temperature*Water Regime on the number of nematodes/g (considering inoculated plants), were tested through an Analysis of Variance of Aligned Rank Transformed Data for a non-parametric factorial analysis [using ART package by Villacorta (2015)].

To assess the nematode effect on A and Ψ_pd_ under the treatment conditions, the variation of these parameters (physiological components related with carbon acquisition and plant water status, respectively) was calculated at each time point by subtracting the respective species control value to each measure (i.e., the difference between the inoculated plants and non-inoculated plants under the same water regime and temperature treatment), with negative values representing lower values compared to the control situation.

All the statistical analyses were conducted using R (R Core Team, 2019).

## Results

Our results showed a distinct physiological performance and severity of the infection among the three *Pinus* species under different temperatures and water regimes.

### The Physiological Impact of Pinewood Nematode on the *Pinus* Species

**FIGURE 2:**
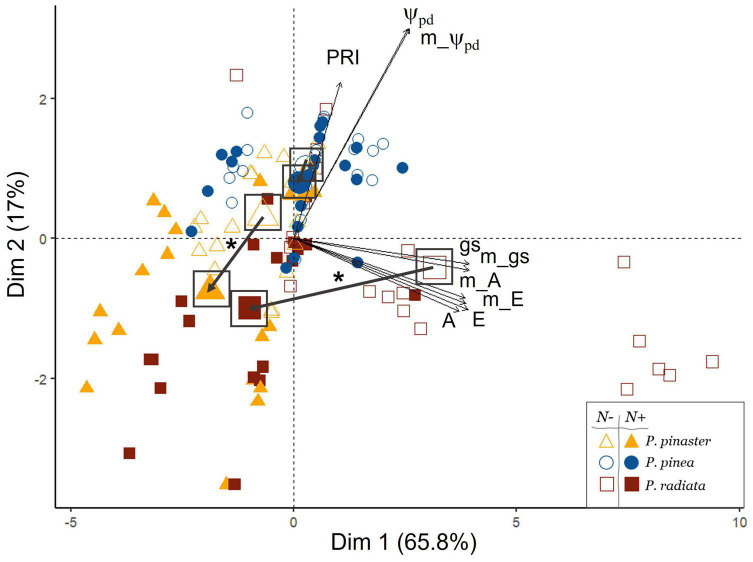
Principal component analysis (PCA) of ecophysiological parameters of the three *Pinus* species, considering the *Bursaphelenchus xylophilus* inoculation treatment (N– non-inoculated/N+ inoculated). Mean values of each group (inoculation treatment by species) are represented by a bigger symbol and highlighted by a square; an arrow represents the bidimensional difference between mean values of each group and an asterisk (*) represents significant differences of PC1 and/or PC2 between groups. Physiological parameters abbreviations: PRI, photochemical reflectance index; Ψ_pd_, water potential predawn; gs, leaf conductance; E, transpiration; A, carbon assimilation; m, minimum.

**FIGURE 3:**
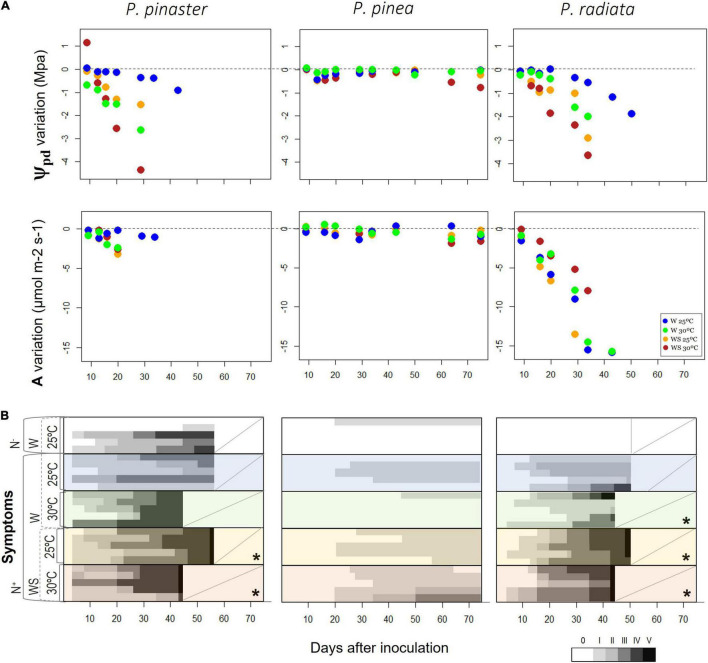
Variation over time of main physiological variables and visible symptoms. **(A)** Pre-dawn water potential (Ψ_pd_) and carbon assimilation (A) from the controlled abiotic conditions to the respective inoculated treatment, i.e., the difference of Ψ_pd_ and A between the inoculated treatments and the associated control (W25: N– vs. N+, WS25: N– vs. N+, W30: N– vs. N+, WS30: N– vs. N+) and **(B)** wilting symptoms after inoculation. Classification of wilting symptoms based on six scaled classes: 0 – tree without symptoms; I – <10% brown leaves; II – 10–50% brown leaves; III – 50–80% brown leaves; IV – >80% brown leaves; V – dead tree without leaves (Proença et al., 2010). An asterisk represents the presence of severe symptoms, with plants reaching death. A diagonal line indicates that the trial was finished, and the trees were removed. See section “Materials and Methods” for detailed information about each treatment and respective abbreviations.

Through the multivariate approach, we found a differentiation among species and inoculation treatments. The first two axis of the PCA (PC1 and PC2) accounted for 82.8% of the total variance (65.8 and 17%, respectively, Figure 2 and Supplementary Table 1). PC1 represented a physiological gradient related to gas exchange and photosynthetic activity, showing increasing values of carbon assimilation (A), transpiration (E), and stomatal conductance (gs), whereas PC2 was mainly reflecting predawn water potential (Ψ_pd_). A significant effect of the interaction species × nematode infection on PC1 was found (*F*-value = 24; *p* < 0.001; Supplementary Table 2). Accordingly, significant differences were found among the pine species depending on the inoculation treatment, with the nematode inoculation significantly reducing the physiological status of *P. pinaster* and *P. radiata*, but not of *P. pinea* (Figure 2 and Supplementary Figures 1, 2). The *Pinus* species segregated along the physiological axis, with a clear differentiation of *P. radiata*, showing higher values of photosynthetic activity under non-infected conditions and a higher decrease of A and gs under nematode inoculation (Figures 2, 3 and Supplementary Figures 1, 2).

### Influence of Temperature and Drought on the Severity of the Pinewood Nematode Infection

Only *P. pinaster* and *P. radiata* overall photosynthetic performance were negatively influenced by the nematode, regardless of their temperature and water regime, as no interaction effect was found for any of the species (Table 1 and Supplementary Figure 4). Alongside, WS plants from all pine species presented lower gas exchange rates and lower Ψ_pd_, indicating a common physiological response to water stress (Supplementary Figures 1, 2).

**TABLE 1:** Effects of temperature (25°C/30°C), water regimes (W – watered/WS – water stressed), *Bursaphelenchus xylophilus* inoculation (N− non-inoculated/N+ inoculated) and their interaction on PC1 values extracted from first PCA axis, and on pre-dawn Water Potential (Ψ_pd_).

		*P. pinaster*				*P. pinea*				*P. radiata*		
						
PC1 (GAS EXCHANGE)	Df	SSq	*F*-value	*p*-value		SSq	*F*-value	*p*-value		SSq	*F* value	*p*-value
Nematode inoculation	1	1254.4	10.01	0.003**		136.9	0.846	0.365		2160.9	22.53	0.000***
Temperature	1	980.1	7.24	0.011*		84.1	0.517	0.478		12.1	0.07	0.787
Water regime	1	1587.6	13.80	0.001***		4000	97.146	0.000***		1562.5	14.02	0.001***
Nematode inoculation: temperature	1	291.6	1.87	0.181		6.4	0.039	0.845		14.4	0.09	0.768
Nematode inoculation: water regime	1	193.6	1.22	0.278		10	0.060	0.807		202.5	1.27	0.267
Temperature: water regime	1	136.9	0.86	0.360		40	0.244	0.625		48.4	0.30	0.587
Nematode inoculation: temperature: water regime	1	90	0.56	0.458		0.90	0.005	0.942		0.9	0.01	0.941
Residuals	32											
**Predawn water potential (**Ψ_pd_**)**												
Nematode inoculation	1	864.0	49.46	0.000***		505.2	17.70	0.001***		958.09	52.08	0.000***
Temperature	1	816.7	40.06	0.000***		440.6	12.79	0.003**		712.97	23.64	0.000***
Water regime	1	864	49.46	0.000***		746.8	45.05	0.000***		964.52	52.32	0.000***
Nematode inoculation: temperature	1	682.7	23.57	0.000***		3.92	0.06	0.811		237.12	4.25	0.055
Nematode inoculation: water regime	1	48.2	0.73	0.406		165.7	3.15	0.096		621.94	20.57	0.000***
Temperature: water regime	1	54	0.84	0.374		62.75	1.09	0.314		181.72	3.24	0.090
Nematode inoculation: temperature: water regime	1	104.2	1.67	0.214		102.0	1.83	0.196		36.17	0.51	0.484
Residuals	16				15				17			

Data analyzed separated by Pinus species. F-values are reported with the corresponding p-value and significance: *p < 0.05, **p < 0.01, and ***p < 0.001.

Over time, the decrease of Ψ_pd_ in infected plants was more evident in *P. radiata* and *P. pinaster*, while *P. pinea* maintained its Ψ_pd_ values similar to the control plants along with the experiment (Figure 3A and Supplementary Figure 1). A higher drop of Ψ_pd_ was observed in infected *P. radiata* and *P. pinaster* under 30°C and water stress (Figure 3A). During the experiment, the highest differences of A compared to their respective control plants were observed in *P. radiata* under water stress. *Pinus pinaster* reached values below −4 MPa only when inoculated with the nematode (Supplementary Figure 1), and the highest decrease was observed in the infection treatment under water and temperature treatments (with a relative decrease of 4 MPa, reaching values of −8 MPa by the end of the experiment; Figure 3A and Supplementary Figure 1). These observed low Ψ_pd_ values could then be linked to the development of foliar damage and earlier tree mortality compared to the other species (Figure 3). Symptom development was different among the pine species, as *P. pinaster* and *P. radiata* plants exhibited PWD severe symptoms, while *P. pinea* plants showed no severe symptoms (Figure 3B and Supplementary Figure 5). In *P. pinaster* and *P. radiata*, the progressive severity of the infection was higher under water stress, and tree mortality was observed as earlier as 43 days after inoculation in WS plants under higher temperatures (Figure 3B).

**FIGURE 4:**
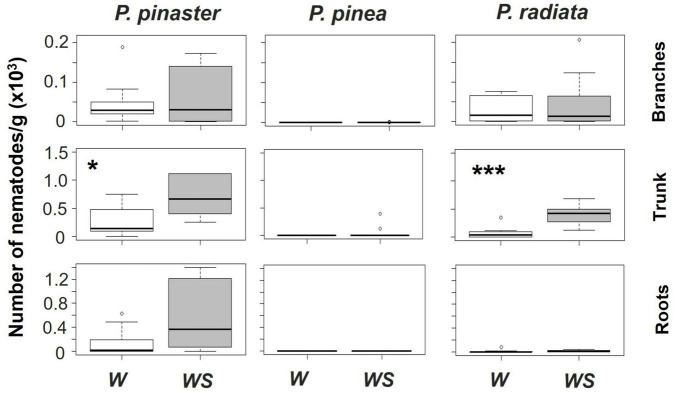
The number of *Bursaphelenchus xylophilus*/g of plant biomass at specific components of the plant (branches, trunk or roots) by *Pinus* species and water regime (W – watered, WS – water stressed), considering both temperature treatments. Significant differences between water regimes within each species are represented by an asterisk (*p < 0.05 and ***p < 0.001). Note that *y*-axis has different scales.

Associated with these symptoms, proliferation of the nematode was verified in the trunk and branches of both *P. pinaster* and *P. radiata* (Figure 4 and Supplementary Table 3). The highest number of nematodes was found in their trunks with water stress promoting nematodes proliferation (no significant effect of the interaction Temperature*Water Regime on nematode number was found, Supplementary Table 4). Moreover, *P. pinaster* was the species with a higher density of nematodes and the only one holding an expressive number of individuals in the roots (Figure 4). Contrastingly, most *P. pinea* plants presented zero nematodes at the end of the experiment (Figure 4).

## Discussion

Susceptibility to PWN was found to be dependent on pine species, being infected *P. pinaster* and *P. radiata* more prone to physiological damages and morphological severe symptoms than *P. pinea*. The impact of nematode inoculation on *P. pinaster* and *P. radiata* was extremely notable, as nematode presence led to a quick physiological performance decrease and to crown discoloration. On the contrary, *P. pinea* expressed almost no wilting symptoms throughout the experiment and was able to keep their physiological performance when infected (Figures 2, 3 and Supplementary Figure 1). Remarkably, *P. pinea* showed to be able to reduce the nematode infection to numbers close to zero even under stressful conditions such as high temperatures and low water availability (Figure 4). These results reinforce that *P. pinea* is the most resistant species to PWN and *P. pinaster* and *P. radiata* are more susceptible to the pathogen.

*Pinus pinea* resistance to PWN is known to be linked to its drought-resistance strategies, generally associated with slow-growing rates and high investment in constitutive defenses (Awada et al., 2003; Tapias et al., 2004; Endara and Coley, 2011). Specifically, the higher concentration of phenolics and tannins, which *P. pinea* can produce, strongly contributed to its lower susceptibility to PWN, compared to the two faster-growing species, *P. pinaster* and *P. radiata* (Tapias et al., 2004; Pimentel et al., 2017). Importantly, our results indicate that the presence of nematode was the main driver of photosynthetic responses in both *P. pinaster* and *P. radiata*, regardless of their temperature or water regime (Figure 2 and Table 1). This result highlights the impact that the nematode has on gas-exchange rates, particularly on *P. radiata*, independently of plants’ temperature or water conditions. It supports the idea that PWN infection promotes a significant and continuous decline in photosynthetic and transpiration rates (Woo et al., 2010), especially in the fast-growing and acquisitive but less chemically defended pines. Generally, fast-growing species must maintain high C uptakes through high rates of carbon assimilation at the cost of decreasing midday leaf water potential (Attia et al., 2015) to promote biomass production. Compared to *P. pinaster*, *P. radiata* showed to be a species with considerably high rates of gas exchange under non-inoculated conditions and showed a stronger gas exchange regulation and decline of carbon assimilation under the presence of the nematode (Figures 2, 3 and Supplementary Figure 1). Thus, *P. radiata* is the most impacted on gas-exchange processes by the presence of the nematode, and through A rate decline measures, the PWN infection could be early detectable in this species.

Nevertheless, it is important to look at other vitality attributes such as plants’ water status. Nematode presence negatively affected Ψ_pd_ of all the pine species, being *P. pinaster* and *P. radiata* the most affected with a significant decline of their water status (Table 1, Figure 3, and Supplementary Figure 1). In this case, the synergy of water stress and PWN infection and the interaction of temperature and nematode infection played a relevant role in the significant decrease of the water status of *P. radiata* and *P. pinaster*, respectively. Thus, even though there was no enhancement of the nematode effect with stress climatic conditions on the photosynthetic performance of these species, the interaction of climatic stressful conditions and PWN-enhanced negative impacts on plants’ water status. The low Ψ_pd_ values observed for these species exposed their incapacity of nighttime recovery through water refilling and their higher susceptibility to xylem cavitation when infected under stressful environmental conditions. The anatomical deterioration of PWN infected trees can more easily permit xylem cavitation through the increase of water tension and/or the increase of vulnerability with the denaturation of living cells in the xylem (Fukuda et al., 2007; Umebayashi et al., 2011) which is exacerbated by water limitation in *P. radiata* and water stress and high temperatures in *P. pinaster*. The dysfunctional water conduction in the xylem embraces a series of physiological alterations such as leaf water potential (Fukuda, 1997; Suzuki, 2002; Menéndez-Gutiérrez et al., 2018; Yazaki et al., 2018), transpiration, and photosynthesis decrease (Fukuda, 1997; Suzuki, 2002; Yazaki et al., 2018).

High temperatures in *P. pinaster* and low water availability in *P. radiata* are therefore expected to further increase the negative impact of the nematode presence through hydraulic failure (Martínez-Vilalta et al., 2009; Choat et al., 2018). The differential nematode effects, which depends on different abiotic factors, are linked to their resistance strategies and physiological response capacity. On one hand, *P. radiata*, with an isohydric response (Brodribb et al., 2014), promptly closed their stomata accompanied by a sharp decrease of gs and A, in the presence of the nematode and particularly under water-limited conditions (Supplementary Figure 1). However, was not able to maintain high levels of Ψ_pd_ (mean value for W25N−: −1.06 MPa), and the decrease in A was followed by a decrease in Ψ_pd_, which denotes a great plant stress affecting both carbon assimilation and water status of the plants (Supplementary Figure 1). Similar to other pine species (such as *Pinus halepensis* and *Pinus edulis*), there are response mechanisms involving carbon starvation from low assimilation and transpiration rates (Skelton et al., 2015; Birami et al., 2018; Breshears et al., 2021) accompanied by hydraulic failure in the shoot. On the other hand, infected *P. pinaster* did not show a great A decrease (through the absence of a strong stomatal control), while losing water, particularly under high temperatures. Stressful temperature conditions are known to promote stomatal opening (Feller, 2006), and the increase in leaf temperatures may decouple net photosynthesis from stomatal conductance, as seen in *Pinus taeda* (Urban et al., 2017). Also, *P. pinaster* is known to have a low genetic variation of cavitation resistance between climatically contrasting populations and very limited phenotypic plasticity (Lamy et al., 2013), conferring this species with low capacity to enable short-term acclimatization and adaptive plasticity to deal with the infection under high temperature and low water availability. *Pinus pinaster* experienced a great decline of water potential (Figure 3A and Supplementary Figure 1), reaching values known to be indicative of high vulnerability to xylem cavitation in *P. pinaster*, way beyond the reference values of P_50_ of −3.8 MPa (Martínez-Vilalta et al., 2009; Corcuera et al., 2011; Bouche et al., 2016), when inoculated with the nematode. The low values of Ψ_pd_ observed in infected plants when under water limitation or high temperature, further associated with unhealthy symptoms (Figure 3B) denotes a process of hydraulic failure occurring under these conditions (Martínez-Vilalta et al., 2009; Choat et al., 2018). This species is therefore highly prone to hydraulic failure, rather than to carbon starvation, when infected by the nematode under high temperatures.

Importantly, associated with the physiological decline of both PWN infected *P. pinaster* and *P. radiata* under drought, we observed severe wilting symptoms, culminating in tree mortality (Figure 3). Under water stress and high temperatures, there was a faster onset of the wilting symptoms (and mortality) compared to no water limitation and mild temperature. Water deficiency is considered a key factor in tree mortality (Futai, 2013; Anderegg et al., 2015a) with morphological symptoms, like the browning of needles, as one of the main consequences. It is expected that xylem cavitation has started before a significant decrease in needle water potential, as also reported in *Pinus thunbergii* (Fukuda et al., 2007) and water shortages affected the progression of disease symptoms because water stress of the trees decreased its tolerance to the disease (Yazaki et al., 2018). As the decline in water status was observed in both affected species, Ψ_pd_ represented a physiological trait more sensitive to environmental conditions (extra exogenous plant stressors), with the impact of the infection further decreasing trees’ water status under higher temperatures (*P. pinaster*) or water limitation (*P. radiata*). As this physiological response anticipated visible symptoms in needles (discoloration), early observations of plants water status (through for example water potential measurements) could serve as potential physiological indicators for an early detection of PWN infected trees, mitigating the decline of Portuguese pine forests observed in recent years (Futai, 2008; Institute for Nature Conservation and Forests [ICNF], 2019).

The total number of nematodes and their allocation on species structure was also different among the pine species and influenced by water limitation (Figure 4). *Pinus pinaster* had the most nematode density and migration to several components of the plants, followed by *P. radiata* and in last *P. pinea*. Besides the lower susceptibility of *P. pinea* to PWN, our results emphasize an intrinsic resistance to this pathogen. Only trees under 30°C and WS were still infected by the end of the experiment, and even in this scenario, nematode populations decreased considerably. This response is likely linked to *P. pinea* anatomy of resin canals (Son et al., 2015) and to the production of constitutive defenses (Pimentel et al., 2017). The most negatively affected species (*P. pinaster* and *P. radiata*) displayed a higher density of nematodes on the main trunk and a proliferation to the branches. Additionally, in *P. pinaster* individuals it was verified a proliferation of nematodes from trunk to roots which could be linked to specific traits such as wider axial resin canals (Nunes da Silva et al., 2015). Proliferation and fast dispersion of nematodes are connected to the weakening and death of seedlings and young trees (Futai, 2013) with distinct species having different tolerances (Nunes da Silva et al., 2015). Furthermore, nematodes’ proliferation of both susceptible species was significantly improved not only by water limitation but also by time course, resulting in a larger number of nematodes in water-stressed plants. In accordance with other studies, water limitation has a crucial role in *B. xylophilus* dynamics (proliferation and migration within tree host) (Fukuda et al., 2007; Futai, 2013; Yazaki et al., 2018), which will consequently have a strong influence on the development of the observed wilting symptoms. Moreover, variation in PWN isolates’ reproductive ability in *Pinus* spp. and their virulence can occur (e.g., significant higher numbers of nematodes after artificial inoculations are excepted for virulent isolates when compared with avirulent PWN isolates) (Aikawa and Kikuchi, 2007; Filipiak, 2015; Cardoso et al., 2022). Thus, intraspecific PWN variability (and the existence of intraspecific variability in the virulence level of certain PWN isolates) might have implications on the effects observed. Nevertheless, we show that the synergy between PWN infection and abiotic conditions, particularly water stress, is relevant for the development of PWD in *P. pinaster* and *P. radiata*.

## Conclusion

The negative impact of the presence of the nematode on physiological traits is observed in both *P. pinaster* and *P. radiata*. *Pinus pinea* showed to be able to resist PWN even under the treatments of higher temperatures and lower water. The presence of the nematode has a particularly strong negative effect on the gas-exchange rates of *P. radiata* and the PWN effect on this species’ water status is exacerbated by water limitation. Additionally, *P. pinaster* water status is significantly affected by PWN under high temperatures. The extra influence of these abiotic factors is also felt on nematode proliferation, on the visible wilting symptoms, and on higher mortality for both species. Our results suggest that, although through different physiological mechanisms (mostly hydraulic failure in *P. pinaster*, and both hydraulic failure and carbon limitation in *P. radiata*), drought increases the susceptibility of these species to PWN. Moreover, this study suggests that the decrease of vitality and mortality due to PWN in *P. pinaster* and *P. radiata*’s can be exacerbated by climate, with the spread of PWD in pine forests of the Mediterranean regions being possibly facilitated by predicted drought conditions (i.e., high temperatures and low water availability). Apart from having different abiotic requests (Keeley, 2012; Vergarechea et al., 2019), the studied pine species showed distinct physiological susceptibilities to PWN, and its species-specific response to PWN under particular environmental pressures are essential and should be considered when defining sustainable forest management strategies.

## Data Availability Statement

The raw data supporting the conclusions of this article will be made available by the authors, upon request to the corresponding author.

## Author Contributions

ME and CA: formal analysis, investigation, and writing – original draft, review, and editing. SC: formal analyses and writing – original draft, review, and editing. AM: laboratory work and writing – review and editing. FC: laboratory work and writing – original draft. IA and LF: conceptualization, laboratory work, investigation, and writing – review and editing. PF and CC: field and laboratory work and writing – original draft. CM and OC: conceptualization, investigation, and writing – review and editing. All authors contributed to the article and approved the submitted version.

## Conflict of Interest

FC was employed by company Infarm. The remaining authors declare that the research was conducted in the absence of any commercial or financial relationships that could be construed as a potential conflict of interest.

## Publisher’s Note

All claims expressed in this article are solely those of the authors and do not necessarily represent those of their affiliated organizations, or those of the publisher, the editors and the reviewers. Any product that may be evaluated in this article, or claim that may be made by its manufacturer, is not guaranteed or endorsed by the publisher.
