# Supplementary material for: Differential Impact of the Pinewood Nematode on Pinus Species Under Drought Conditions
Source: Front Plant Sci. 2022 Mar 10;13:841707. doi: 10.3389/fpls.2022.841707 (PMC8961127; doi:10.3389/fpls.2022.841707)
Supplement: Supplementary file 1 [file Data_Sheet_1.pdf]

Supplementary Material

Supplementary Table S1. Summary of principal component analyses (PCA) of the monitored ecophysiological parameters (PRI – Photochemical Reflectance Index, A- Carbon assimilation, E – Transpiration, gs - Leaf conductance, Ψ_{pd} – pre-dawn water potential) including mean and minimum values. Respective Eigenvalues and variance explained by the Dimension 1 and 2 are represented in the lower part of the table.

	**Dim.1**			**Dim.2**		
	**Coordinates**	**ctr**	**cos2**	**Coordinates**	**ctr**	**cos2**
MeanWP	0.62	6.479	0.384	0.715	33.367	0.511
MinWP	0.612	6.332	0.375	0.714	33.309	0.51
MeanA	0.885	13.207	0.782	-0.248	4.003	0.061
MinA	0.918	14.227	0.843	-0.201	2.645	0.04
Meangs	0.937	14.815	0.878	-0.107	0.751	0.011
Mings	0.94	14.908	0.883	-0.085	0.475	0.007
MeanTransp	0.933	14.699	0.871	-0.242	3.825	0.059
MinTransp	0.92	14.281	0.846	-0.22	3.175	0.049
PRI	0.25	1.053	0.062	0.531	18.45	0.282
**Eigenvalue**		5.924			1.53	
**Variance %**		65.828			17.004	
**Cumulative (Var %)**		65.828			82.832	

Supplementary Table S2. Effects of temperature (25ºC/30ºC), water regime (W – Watered/WS – Water Stressed), *Bursaphelenchus xylophilus* inoculation (N- non inoculated / N+ inoculated), *Pinus* species (*P. pinaster, P. pinea, P. radiata*) and their interaction on PC1 and PC2 values. Significant F-values are reported with the corresponding P value and significance value: * p<0.05, ** p<0.01 and *** p<0.001.

			PC1		PC2	
**Treatment**	**Df**	**Df res**	**F-value**	**p-value**	**F-value**	**p-value**
Species	2	96	32.75	0.000***	54.37	0.000***
Temperature	1	96	4.30	0.041*	17.25	0.000***
Water Regime	1	96	93.99	0.000***	76.50	0.000***
Inoculation	1	96	61.24	0.000***	28.64	0.000***
Species x Temperature	2	96	2.14	0.123	0.19	0.824
Species x Water regime	2	96	3.23	0.044*	5.86	0.004**
Temperature x Water regime	1	96	0.00	0.996	0.05	0.817
Species x Inoculation	2	96	24.04	0.000***	4.65	0.012*
Temperature x Inoculation	1	96	1.09	0.298	4.31	0.041*
Water regime x Inoculation	1	96	0.25	0.615	12.08	0.001***
Species x Temperature x Water regime	2	96	0.73	0.482	0.20	0.818
Species x Temperature x Inoculation	2	96	0.57	0.568	0.29	0.749
Species x Water regime x Inoculation	2	96	1.84	0.165	4.02	0.021*
Temperature x Water regime x Inoculation	1	96	0.34	0.563	0.60	0.440
Species x Temperature x Water regime x Inoculation	2	96	0.23	0.797	0.25	0.777

Supplementary Table S3. Mean±standart deviation of biomass (branches, trunk, or roots) of *Pinus pinaster* and *P. radiata* at the end of the experiment, and respective number of *Bursaphelenchus xylophilus* (Bx)/g. The total number of *B. xylophilus* by species and treatment (temperature: 25ºC/30ºC, water regimes: W – Watered / WS – Water Stressed) was estimated based on plants biomass and the number of *B. xylophilus/g.*

			**Branch**		**Trunk**		**Root**		**Total**		
***Pinus* spp.**	**Treatment**		**Biomass (g)**	**Bx/g**	**Biomass (g)**	**Bx/g**	**Biomass (g)**	**Bx/g**	**Biomass (g)**	**Bx/g**	**Total Bx (estimated)**
Pinus pinaster	**W**	**25**	50.3±43	49±79	328.1±136	55±79	226.7±22	9±10	605.2±187	114±143	68940±26770
		**30**	41.2±12	25±23	322.9±127	452±244	238.7±35	264±145	602.8±155	741±446	446586±68986
	**WS**	**25**	35.8±26	104±76	261.3±125	1119±877	178.3±56	447±276	475.4±252	1670±1391	793685±350407
		**30**	38.1±33	22±37	322.7±150	800±851	205.1±56	680±310	565.9±222	1501±933	849624±207545
Pinus radiata	**W**	**25**	5.9±2	110±205	99.1±17	95±148	71.9±12	1±1	177.0±29	206±352	36369±10087
		**30**	7.0±4	32±36	94.0±30	49±39	43.5±8	22±16	144.5±45	104±63	14987±2826
	**WS**	**25**	4.7±3	81±86	94.2±53	515±173	57.3±8	8±5	156.2±71	605±152	94444±10813
		**30**	4.7±1	9±9	69.7±15	317±132	48.3±12	19±14	122.6±34	345±149	42249±5103

Supplementary Table S4. Effects of Temperature (25ºC/30ºC), water regimes (W – Watered / WS – Water Stressed), and its interaction on the number of *Bursaphelenchus xylophilus*/g. Data analyzed for specifics components of the plant (branches, trunk, or roots) and separated by *Pinus* species (*P. pinaster, P. pinea, P. radiata*). F-values are reported with the corresponding p-value and significance: * p<0.05, ** p<0.01 and *** p<0.001.

	P. pinaster			*P. pinea*			*P. radiata*		
**Branch**	**Df**	**F-value**	**p-value**	**Df**	**F-value**	**p-value**	**Df**	**F value**	**p-value**
Temperature	1	5.492	0.032 *	1	3.464	0.081	1	1.012	0.329
Water Regime	1	0.318	0.581	1	3.464	0.081	1	0.006	0.939
Temperature x Water Regime	1	0.864	0.366	1	3.464	0.081	1	0.379	0.547
**Trunk**									
Temperature	1	0.054	0.820	1	3.478	0.081	1	4.186	0.058
Water Regime	1	14.198	0.002 **	1	3.478	0.081	1	37.161	0.000 ***
Temperature x Water Regime	1	3.227	0.091	1	3.478	0.081	1	1.549	0.231
**Roots**									
Temperature	1	1.016	0.328		-	-	1	2.555	0.130
Water Regime	1	3.743	0.071		-	-	1	0.144	0.709
Temperature x Water Regime	16	0.079	0.782		-	-	16	0.000	1.000

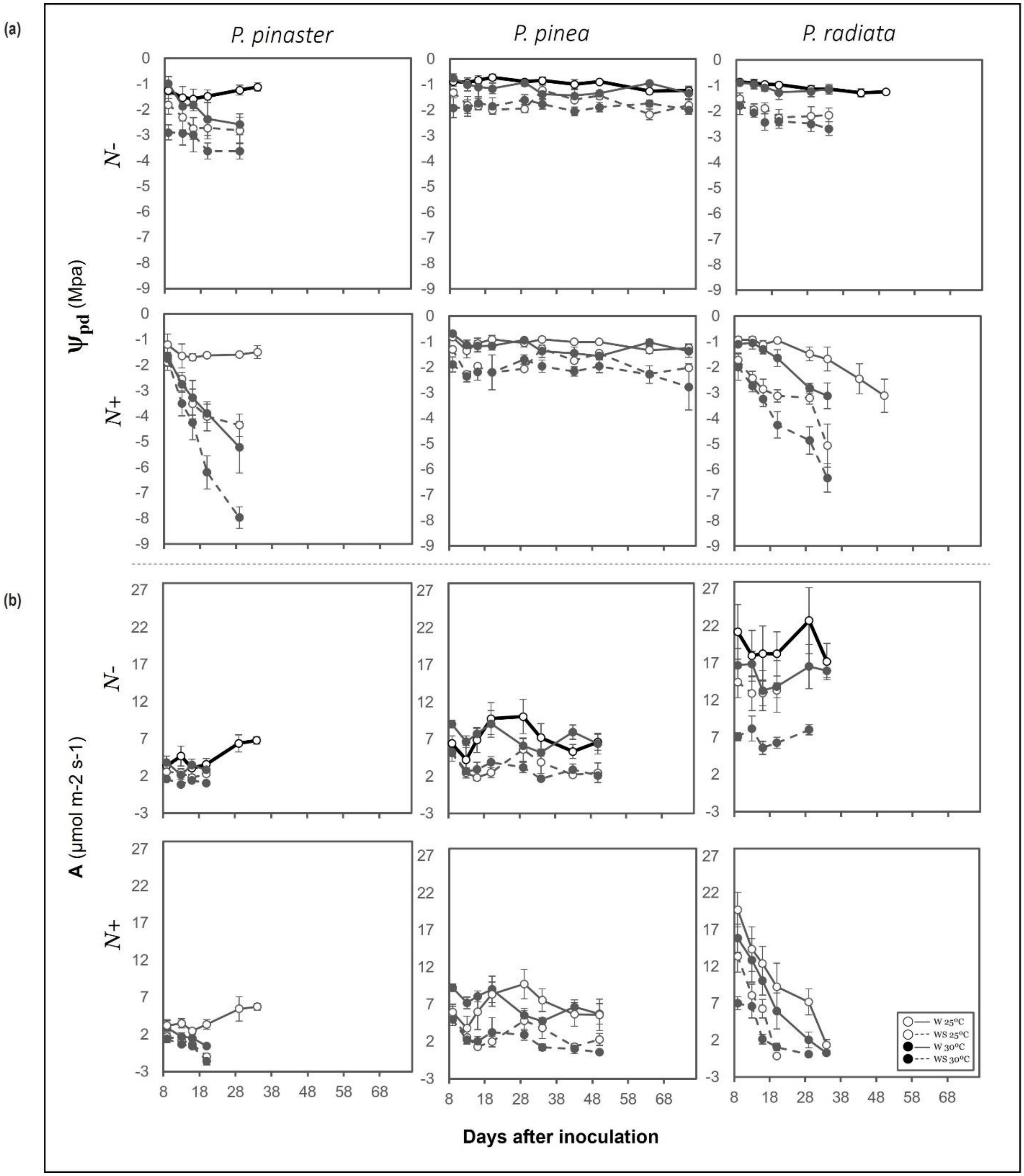

Supplementary Figure S1. Monitorization of (a) pre-dawn water potential (Ψ_{pd}) and (b) carbon assimilation (A) by treatments [temperature (25ºC/30ºC); water regimes (W – Watered / WS – Water Stressed); *Bursaphelenchus xylophilus* inoculation (N- non inoculated/N+ inoculated)] for the three *Pinus* species (*P. pinaster*, *P. pinea* and *P. radiata*). Mean values and respective standard deviation for each sampling day were considered.

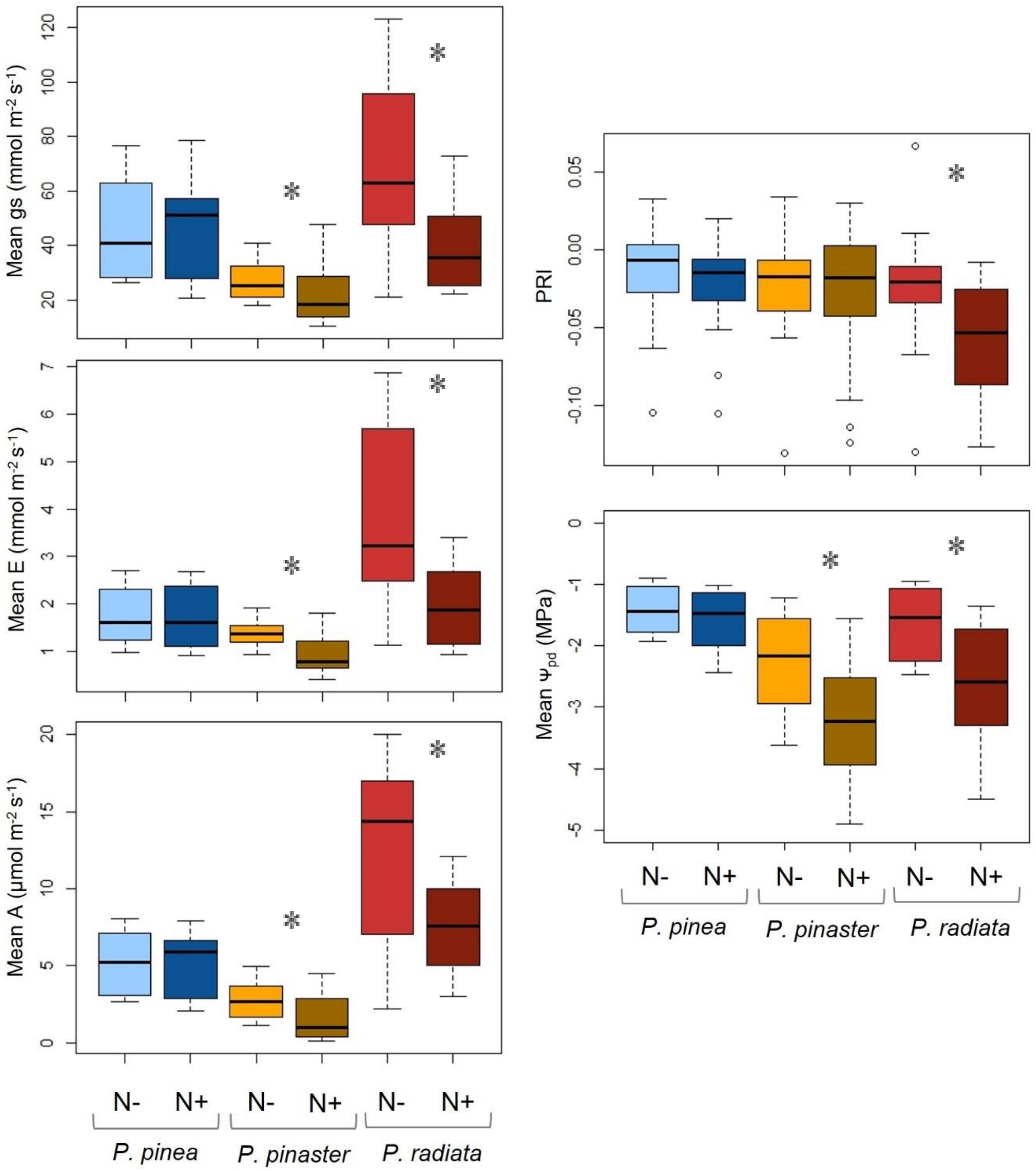

Supplementary Figure S2. Physiological measurements grouped by *Pinus* species and *Bursaphelenchus xylophilus* inoculation (N- non inoculated/N+ inoculated), considering both temperatures treatments and water regimes. Significant differences (p-value<0.05) between inoculation treatments within each species are represented by an asterisk (assessed by mixed models with Resp ~ TreatN as fixed effects and time as a random effect (~1 | DaysAfterInoc). Physiological parameters: PRI – Photochemical Reflectance Index (measured once 15 days after inoculation), A- Carbon assimilation, E – Transpiration, gs - Leaf conductance, Ψ_{pd} – pre-dawn water potential (mean values of all measures collected during the trial).

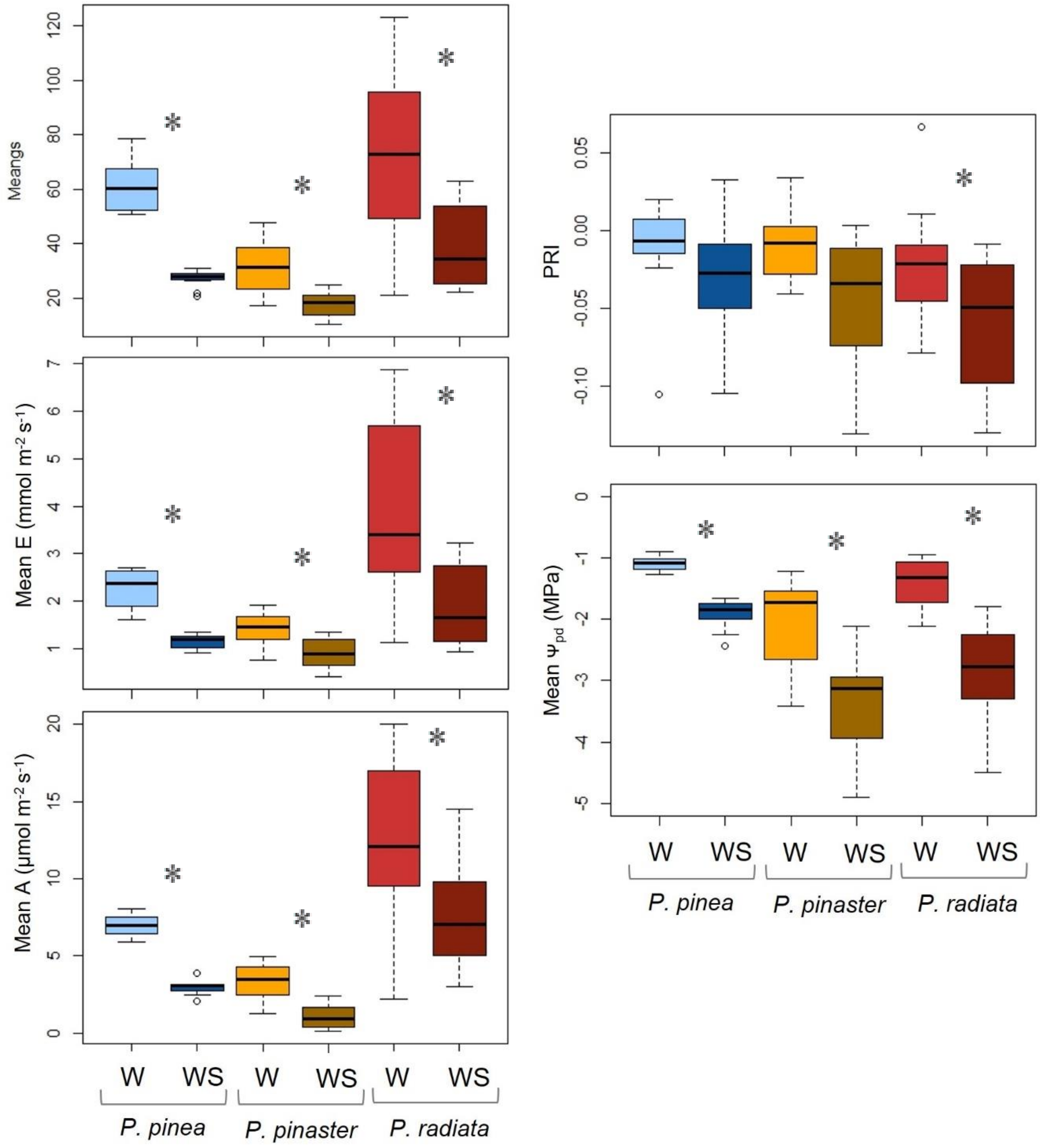

Supplementary Figure S3. Physiological measurements grouped by *Pinus* species and water regime (W – Watered/WS – Water Stressed), considering both temperatures and *Bursaphelenchus xylophilus* inoculation treatments. Significant differences (p-value<0.05) between water regime treatments within each species are represented by an asterisk (assessed by mixed models with Resp ~ TreatW as fixed effects and time as a random effect (~1 | Days After Inoculation). Physiological parameters: PRI – Photochemical Reflectance Index (measured once 15 days after inoculation), A- Carbon assimilation, E – Transpiration, gs - Leaf conductance, Ψ_{pd} – pre-dawn water potential (mean values of all measures collected during the trial).

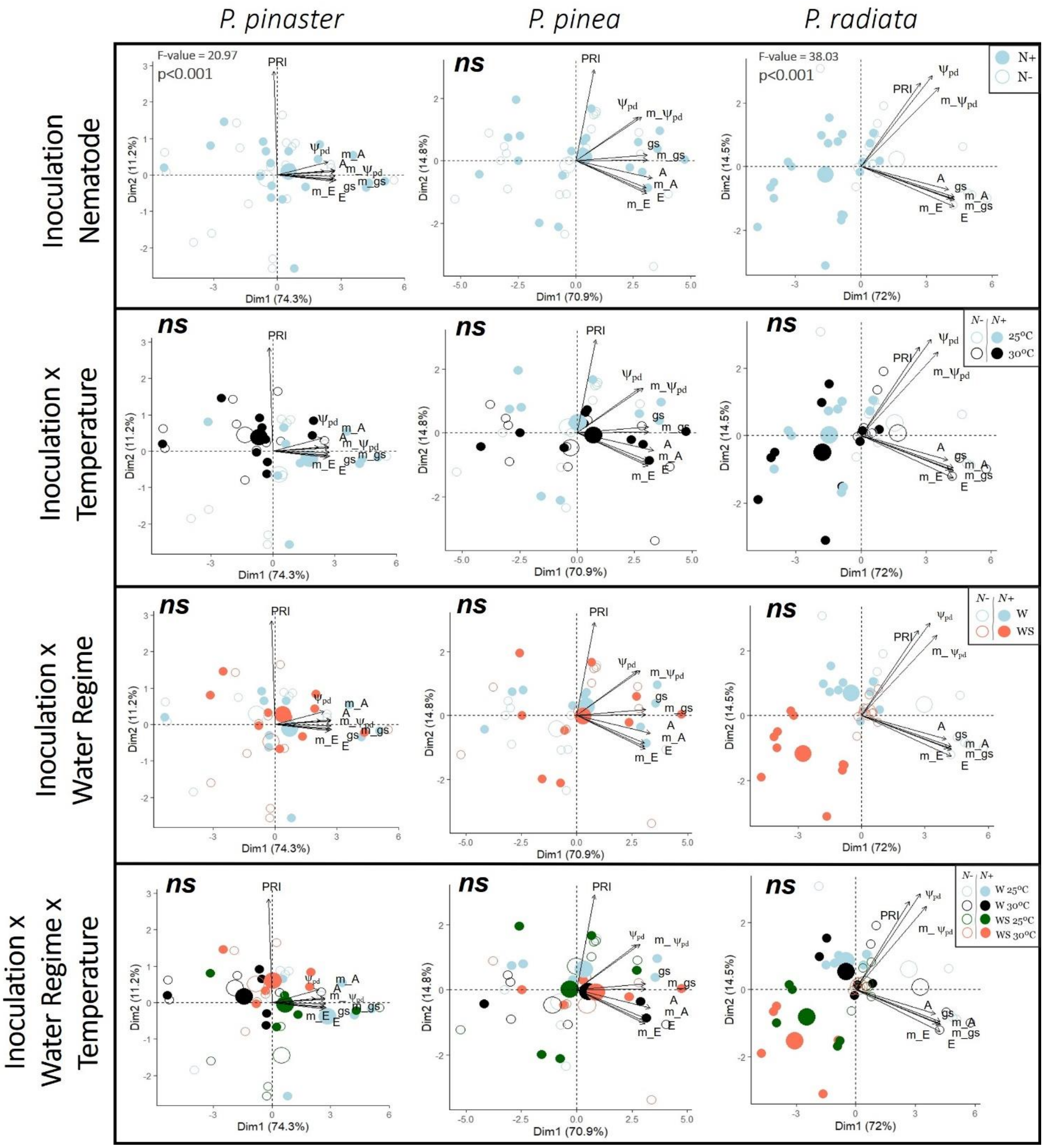

Supplementary Figure S4. Principal Component Analysis (PCA) of physiological parameters of *Pinus* species based on inoculation, temperature, and water regime) and its integration. Physiological parameters considered: PRI -Photochemical Reflectance Index; Ψ_{pd} – Water potential predawn, minimum, and mean; gs – leaf conductance, minimum and mean; E – transpiration, minimum and mean; A – Carbon assimilation, minimum and mean. See inner legend for species and treatment symbology. Significant effects of the treatment on PC1 are reported with respective F-values and P- value, ns stands for non-significant effects.

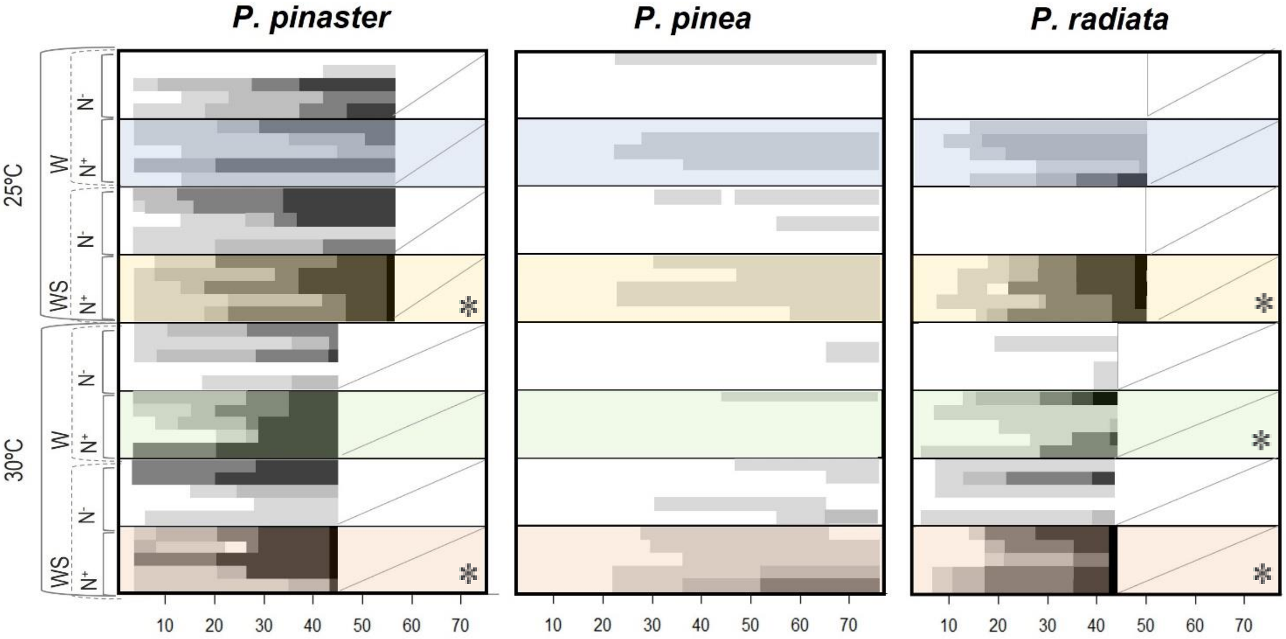

Supplementary Figure S5. Classification of visible wilting symptoms after inoculation based on six scaled classes: 0 – tree without symptoms; I - <10% brown leaves; II - 10–50% brown leaves; III – 50–80% brown leaves; IV - >80% brown leaves; V- dead tree without leaves (Proença et al. 2010). The treatments highlighted with colors were inoculated with *Bursaphelenchus xylophilus (Blue:W25N+, Yellow: WS25N+, Green: W30N+, Red: WS30N+) and black and white situations correspond to control treatments (W25N-, WS25N-, W30N-, WS30N-).* An asterisk represents the presence of severe symptoms, with plants reaching death. A diagonal line indicates that the trial was finished, and the trees were removed. See methods section for detailed information about each treatment and respective abbreviations.
